# Examining putamen resting-state connectivity markers of suicide attempt history in depressed adolescents

**DOI:** 10.3389/fpsyt.2024.1364271

**Published:** 2024-06-06

**Authors:** Olga Tymofiyeva, Tiffany C. Ho, Colm G. Connolly, Sasha Gorrell, Ryan Rampersaud, Sabrina M. Darrow, Jeffrey E. Max, Tony T. Yang

**Affiliations:** ^1^ Department of Radiology and Biomedical Imaging, University of California San Francisco, San Francisco, CA, United States; ^2^ Department of Psychology, Brain Research Institute, Interdepartmental Graduate Program in Neuroscience, University of California, Los Angeles, Los Angeles, CA, United States; ^3^ Department of Biomedical Sciences, Florida State University College of Medicine, Tallahassee, FL, United States; ^4^ Department of Psychiatry and Behavioral Sciences, Division of Child and Adolescent Psychiatry, University of California San Francisco, San Francisco, CA, United States; ^5^ Department of Psychiatry, University of California San Diego, San Diego, CA, United States; ^6^ Department of Psychiatry, Rady Children’s Hospital, San Diego, CA, United States; ^7^ Weill Institute for Neurosciences, University of California San Francisco, San Francisco, CA, United States

**Keywords:** adolescent, suicide, resting state connectivity, functional magnetic resonance imaging, putamen

## Abstract

**Introduction:**

Suicide is a current leading cause of death in adolescents and young adults. The neurobiological underpinnings of suicide risk in youth, however, remain unclear and a brain-based model is lacking. In adult samples, current models highlight deficient serotonin release as a potential suicide biomarker, and in particular, involvement of serotonergic dysfunction in relation to the putamen and suicidal behavior. Less is known about associations among striatal regions and relative suicidal risk across development. The current study examined putamen connectivity in depressed adolescents with (AT) and without history of a suicide attempt (NAT), specifically using resting-state functional magnetic resonance imaging (fMRI) to evaluate patterns in resting-state functional connectivity (RSFC). We hypothesized the AT group would exhibit lower striatal RSFC compared to the NAT group, and lower striatal RSFC would associate with greater suicidal ideation severity and/or lethality of attempt.

**Methods:**

We examined whole-brain RSFC of six putamen regions in 17 adolescents with depression and NAT (M_Age_ [SD] = 16.4[0.3], 41% male) and 13 with AT (M_Age_ [SD] = 16.2[0.3], 31% male).

**Results:**

Only the dorsal rostral striatum showed a statistically significant bilateral between-group difference in RSFC with the superior frontal gyrus and supplementary motor area, with higher RSFC in the group without a suicide attempt compared to those with attempt history (voxel-wise *p*<.001, cluster-wise *p*<.01). No significant associations were found between any putamen RSFC patterns and suicidal ideation severity or lethality of attempts among those who had attempted.

**Discussion:**

The results align with recent adult literature and have interesting theoretical and clinical implications. A possible interpretation of the results is a mismatch of the serotonin transport to putamen and to the supplementary motor area and the resulting reduced functional connectivity between the two areas in adolescents with attempt history. The obtained results can be used to enhance the diathesis-stress model and the Emotional paiN and social Disconnect (END) model of adolescent suicidality by adding the putamen. We also speculate that connectivity between putamen and the supplementary motor area may in the future be used as a valuable biomarker of treatment efficacy and possibly prediction of treatment outcome.

## Introduction

Suicide is currently a leading cause of death in adolescents and young adults (1). The neurobiological correlates of suicide risk in youth, however, remain unclear (see (2) for a review). Given that adolescence represents a time of significant brain plasticity governing the growth of cognitive and social competencies (3) as well as puberty-related changes (4), clarifying the neural correlates of suicide risk in this vulnerable population will have important implications for developing adolescent-specific interventions to reduce suicidality during this high-risk period.

To prevent adolescent suicide and employ our current assessment methods more effectively, there is an urgent need to know *who* is at risk for attempting suicide and *when* that risk is greatest. However, to date, consensus is lacking for a brain-based model of adolescent suicidal behavior. One of the most recent models has been proposed by Mann and Risk, highlighting neurotransmitter dysregulation, and in particular, consistent evidence established over several decades that deficient serotonin release is a biomarker of suicidal behavior (5–7). With this foundation, there has been increasing interest in forming distinct associations of serotonergic markers with depression versus suicide (8, 9), and with violent versus nonviolent suicide (10, 11). Evidence from positron-emission topography (PET) neuroimaging studies suggests that serotonin dysregulation—and specifically up-regulated serotonin 1A autoreceptor binding in brainstem raphe nucleus neurons—is predictive of higher-lethality suicidal behavior (12, 13).

Notably, this work collectively suggests that although the role of serotonergic dysfunction in mood disorders and suicidality is complex, clarifying specific neural correlates with suicidality can be informed by examination of the putamen. Specifically, serotonin is released by brainstem raphe nuclei into the dorsal striatum which comprises the caudate and putamen (14); here, putamen serotonin levels supersede that of the caudate (15). In one PET study of adults with depression and at least one prior suicide attempt, serotonin binding potential measured via serotonin transporter (SERT) activity was significantly decreased in the midbrain/pons and putamen of patients relative to healthy controls (16). In another PET study of adolescents that probed a peer interaction task, higher suicidal ideation was associated with reduced putamen activity (17). Taken together, this work suggests that it is likely that the putamen plays an important role related to risk of suicide attempts in adolescents, a hypothesis that remains untested. In a study of adult subjects that seeded the putamen during a motor-task paradigm, striatal motor/sensory network connections were associated with almost exclusively suicidal behaviors (with one subject in the study displaying non-suicidal self-harm behavior) (18). Although increasing interest has been devoted to task-based functional magnetic resonance imaging (fMRI) in the study of suicidal behavior in adolescents – both as a safer alternative to PET, and a determinant of functional connectivity associated with suicide risk, it is difficult to pinpoint the precise functional network patterns that are associated with suicidal behavior given the diverse tasks that have been used across investigations. Thus, it is imperative that we investigate patterns of intrinsic (i.e., task-independent) functional connectivity if we are to facilitate comparisons across samples and studies, and to identify specific neurobiological targets with relevance in clinical research studies.

No studies to date have specifically examined resting-state fMRI patterns of the putamen in adolescents with and without a history of a suicide attempt. In the current study, we sought to address this important knowledge gap by comparing whether striatal resting-state functional connectivity (RSFC) differentiated depressed adolescents with a history of a suicide attempt (AT) versus those without (NAT). While not all suicidal adolescents are depressed, we chose to examine a depressed population because of the high risk for suicidal behavior in depressed adolescents. We conducted a comprehensive assessment of history of depression and suicidal thoughts and behaviors using well-validated, interview-based instruments. Critically, our study directly compared depressed adolescents with a history of AT with a well-matched group that was also depressed but with no history of AT; comparisons with a psychiatrically healthy control group would not be sufficient for determining whether the suicide attempt-related RSFC patterns we observe are in fact due to a history of AT or simply the presence of a psychiatric disorder. In addition, we explored whether striatal RSFC patterns were associated with severity of suicidal ideation or lethality of attempt. Based on the studies reviewed above, we hypothesized that: 1) the AT group would exhibit lower striatal RSFC compared to the NAT group, and 2) lower striatal RSFC would be associated with greater suicidal ideation severity and/or lethality of attempt.

## Methods and materials

### Participants

Thirty adolescents (aged 13-17 years; 11 male/19 female) with Major Depressive Disorder (MDD) were recruited from 35 adolescent psychiatric and primary care clinics throughout the San Diego county area (United States). Although multiple clinics referred potentially depressed adolescents to the study, all diagnoses of MDD were made independently of the source clinic and all scanning took place at only one site. Gathering of all self-report measures was conducted at the same site or was self-paced at the participant’s home. All participants were medically healthy and were not taking any antidepressant medications or supplements at the time of scan.

Participants gave written informed assent and their parent/legal guardian provided written informed consent. Participants were financially compensated for their time. The institutional review boards of University of California San Diego, University of California San Francisco, Rady Children’s Hospital, and the county of San Diego approved this study.

### Demographic and clinical assessments

In this paper we define suicidality as suicidal ideation or behavior, and we define suicidal behavior as a completed or uncompleted suicide attempt. To determine diagnostic status, the Schedule for Affective Disorders and Schizophrenia or School-Age Children-Present and Lifetime Version (19) was administered to all adolescents. Depressive symptom severity was assessed using the clinician-administered Children’s Depression Rating Scale-Revised (CDRS-R) (20), the self-reported Beck Depression Inventory-2 (BDI-II) (21, 22) and Reynolds Adolescent Depression Scale-2 (RADS-2) (23). Anxiety was measured using the Multidimensional Anxiety Scale for Children (MASC) (24). Trauma was measured with the Childhood Trauma Questionnaire (CTQ) (25); this measure was added to the assessment battery at a later timepoint, which contributed to expected missing data (reported in **Table 1**). The Stressful Life Events Schedule (SLES) was used to assess stressors in children and adolescents (26). Psychosocial functioning was assessed with the Children’s Global Assessment Scale (CGAS) (27). All participants were also administered the Wechsler Abbreviated Scale of Intelligence (WASI) (28); Standard Snellen Eye Chart (29); Ishihara Color Plates test (8 plate, 2005 ed.) (30); Customary Drinking and Drug Use Record (CDDR) (31); and the Family Interview for Genetics Studies (FIGS) (32). Participants also reported demographics, Tanner Stage, and medical and developmental history.

**Table 1:** Summary of demographic and clinical differences between depressed adolescents with and without a history of attempt.

Characteristic	AT	NAT	Statistic	*p*-value
**Number of participants (n)**	13	17		
**Gender (M/F)**	4/9	7/10	X^2^(1.00) = 0.04	0.84
**Age at time of scan (years)**	16.2 ± 0.3 (14.4-17.8)	16.4 ± 0.3 (14.2-17.9)	t(27.46) = -0.59	0.56
**Hollingshead Socioeconomic Score**	33 ± 15 (11-59)†	26 ± 36 (11-70)†	W = 120	0.71
**Tanner Score**	4.5 ± 0.5 (3-5)†	4 ± 1 (3-5)†	W = 108	0.95
**Wechsler Abbreviated Scale of Intelligence (Verbal)**	111.7 ± 3.5 (89-132)	99.8 ± 2.8 (87-131)	t(24.73) = 2.64	**0.01**
**Wechsler Abbreviated Scale of Intelligence (Performance)**	103.5 ± 3 (86-120)	98.5 ± 2.2 (80-110)	t(23.38) = 1.35	0.19
**Wechsler Abbreviated Scale of Intelligence (Full)**	108.2 ± 3.2 (89-129)	99.4 ± 2 (84-119)	t(20.81) = 2.37	**0.03**
**Children’s Global Assessment Scale**	65 ± 19 (47-85)†	70 ± 25 (48-90)†	W = 112	0.95
**Children’s Depression Rating Scale (Standardized)**	69.3 ± 4 (33-85)	66.7 ± 2.6 (44-82)	t(21.31) = 0.55	0.59
**Reynolds Adolescent Depression Scale Dysphoric Mood (Standardized)**	65 ± 2.3 (47-76)	60.2 ± 3.2 (35-78)	t(26.90) = 1.23	0.23
**Reynolds Adolescent Depression Scale Anhedonia/Negative Affect (Standardized)**	55.3 ± 1.8 (40-65)	53.3 ± 3.5 (15-73)	t(23.56) = 0.51	0.62
**Reynolds Adolescent Depression Scale Negative Self-evaluation (Standardized)**	64.5 ± 3.2 (40-81)	59.3 ± 3.1 (39-85)	t(27.27) = 1.18	0.25
**Reynolds Adolescent Depression Scale Somatic Complaints (Standardized)**	58.7 ± 1.8 (44-67)	57.9 ± 2.8 (31-74)	t(25.95) = 0.23	0.82
**Reynolds Adolescent Depression Scale Total (Standardized)**	64.3 ± 2.6 (42-80)	61.1 ± 3.5 (35-87)	t(27.58) = 0.75	0.46
**Beck Depression Inventory II**	23.2 ± 3.1 (4-45)	22.3 ± 3.5 (0-47)	t(27.99) = 0.20	0.84
**Multidimensional Anxiety Scale for Children (Standardized)**	55.4 ± 2.1 (41-68)	57.2 ± 2.9 (32-76) [1]	t(25.99) = -0.49	0.63
**Childhood Trauma Questionnaire (Total)**	56.4 ± 2.9 (43-70) [4]	51.6 ± 5.2 (40-67) [12]	t(6.58) = 0.81	0.44
**CTQ: Emotional Abuse**	14.4 ± 1.3 (9-19) [4]	11.4 ± 2.7 (5-17) [12]	t(5.95) = 1.03	0.34
**CTQ: Physical Abuse**	8 ± 0.6 (6-12) [4]	6.8 ± 1.1 (5-10) [12]	t(6.40) = 0.95	0.38
**CTQ: Sexual Abuse**	5.3 ± 0.3 (5-8) [4]	5 ± 0 (5-5) [12]	t(8.00) = 1.00	0.35
**CTQ: Emotional Neglect**	13.8 ± 1.5 (7-20) [4]	11.2 ± 3 (5-21) [12]	t(5.94) = 0.77	0.47
**CTQ: Physical Neglect**	8.8 ± 1 (5-12) [4]	8.4 ± 1 (5-11) [12]	t(10.69) = 0.27	0.79
**CTQ: Minimization/Denial**	5.1 ± 0.7 (3-8) [4]	6.8 ± 1.6 (3-11) [12]	t(5.39) = -0.98	0.37
**SLES: Number of stressful events**	10 ± 11 (2-20)†	9 ± 7.5 (1-30) [2]†	W = 114	0.45
**SLES: Number of severe events**	5 ± 8 (1-14)†	3 ± 4.5 (0-27) [2]†	W = 117	0.38
**SLES: Total Sum Stress**	29 ± 37 (4-60)†	19 ± 18 (3-100) [2]†	W = 120	0.31
**C-SSRS: Lethality (median, range)**	1 (0-3)			
**C-SSRS: NSSI (%)**	15.38%	5.88%		
**C-SSRS: SI (median, range)**	5 (1-5)	1 (1-3)		

SD, standard deviation; IQR, interquartile range; M, male; F, female; C-SSRS, Columbia Suicide Rating Scale; NSSI, non-suicidal self-injurious behaviors; SI, suicidal ideation.

Mean ± SD or median ± IQR if indicated by †. [] indicated the number of missing data points.

Statistic: W, Wilcox rank sum test; χ^2^, χ^2^ test for equality of proportions; t, Student’s t-test.Bold means p < .05.

Exclusion criteria were as follows:

1. IQ score < 80, as determined by the WASI.
2. Color blindness or having less than 20/40 correctible vision as established by the Ishihara Color Plates test and Standard Snellen Eye Chart, respectively.
3. Contraindications for MRI (e.g., ferrometallic implants, braces, claustrophobia).
4. Pregnancy or the possibility thereof.
5. Evidence of drug misuse (illicit or prescription) within the previous month or two or more alcoholic drinks per week currently or within the previous month as determined by the CDDR.
6. Left-handedness.
7. Prepubertal status (< Tanner stage 3).
8. Inability to comprehend and comply with study procedures.
9. Use of medications with a central nervous system effect in the two weeks prior to scanning.
10. Any history of neurologic disorder (e.g., meningitis, migraine, HIV), head trauma, a learning disability, serious medical health problems, or a complicated or premature birth before 33 weeks gestation (due to the possibility of abnormal neurodevelopment).
11. CDRS-R T-score < 55.
12. A primary psychiatric diagnosis other than MDD.

### Assessment of history of suicide attempt and ideation

History of suicide attempt was assessed by administering the pediatric version of the Columbia Suicide Severity Rating Scale (C-SSRS) (33), a semi-structured interview that probes lifetime history of suicidal thoughts (including nature and severity of ideation) and behaviors (including preparatory acts, and actual, interrupted, or aborted attempts). For our analyses, any actual, interrupted, or aborted attempt was classified as an AT. We used a 0-5 coding for lethality as a continuous measure of lethality severity. We also summed the five suicidal ideation severity items (lifetime) to compute a continuous measure of suicidal ideation severity. These measures of lethality and suicidal ideation severity were used in subsequent analyses assessing brain-behavior associations within the AT group only.

### MR data acquisition

MR images were acquired on a 3T GE MR750 MRI system (Milwaukee, WI) at the Center for Functional MRI at the University of California, San Diego. One 8 min 32 sec T2*-weighted echo planar image (EPI) scan (256 volumes TR/TE=2s/30ms, flip angle=90°, 64×64 matrix, 3×3×3mm voxels, 40 axial slices, parallel imaging method: ASSET, acceleration factor: 2) was acquired. A T1-weighted (T1w) scan (TR/TE=8.1ms/3.17ms, flip angle=12°, 256×256 matrix, 1×1×1mm voxels, 168 sagittal slices) was acquired for spatial normalization and functional localization. Participants were instructed to lay as still as possible without falling asleep and were presented with a fixation cross that was placed centrally on a screen at the foot of the scanner and viewed via a head coil-mounted mirror. All participants were asked whether they had fallen asleep during the scan, and all adolescents reported that they had not.

### Functional MRI preprocessing

In-house scripts using AFNI (34) and FSL (35) were used for analysis. T1w images were skull-stripped and transformed to MNI152 space using linear (36, 37) and nonlinear (38) alignment. Cerebrospinal fluid (CSF), grey matter (GM), and white matter (WM) were then segmented (39). EPI time-series were slice-time and motion corrected, aligned to the T1w images (40) and smoothed with a 4.2mm full-width at half-maximum isotropic Gaussian kernel within a GM mask.

To control for the effects of physiological processes (cardiac and respiratory cycles) (41) we removed signal associated with several nuisance covariates. Specifically, we regressed from each volume of the EPI time-series signal associated with the six motion parameters, mean signal from the ventricles, a local estimate of the signal from white matter, and their de-trended derivatives (i.e., 16 regressors of non-interest). Local white matter regressors were created for each voxel within the eroded white matter mask by averaging the signal within a local spherical mask (5mm radius) around each voxel. This method permits the estimation of the nuisance parameters while simultaneously avoiding the grey matter. Additionally, it has been shown to be robust to distance-dependent motion artifacts in resting state analysis (42). We opted not to include a global signal regressor due to the controversy surrounding its use for connectivity-based analyses (43–47). Band-pass filtering (0.009 – 0.08 Hz) was conducted simultaneously on the EPI data and nuisance regressors to avoid the reintroduction of signal outside the range of the band-pass filter during the subsequent multiple linear regression process (48).

Finally, since motion can produce spurious correlations in resting-state data, we adopted the volume scrubbing technique advocated by Power et al. (49). The scrubbing procedure identifies and censors volumes that exhibit abnormal values in movement-related emetrics. This technique relies on two movement-related metrics: frame-wise displacement (FD) and the temporal derivative of the root mean square (RMS) variance over voxels (DVARS). Here, volumes were excluded if FD exceeded 0.5 or the DVARS exceeded 5. Additionally, the volume immediately preceding, and 2 volumes immediately after each censored volume were also excluded. This was combined with the removal of outlier volumes where more than 10% of voxels were greater than the median absolute deviation of the detrended time-series. Participants who had more than 20% (50 volumes) of their volumes censored were excluded from further analysis.

The cleaned time-series were transformed to MNI152 standard space at 3×3×3mm resolution for subsequent analyses.

### Seed creation and RSFC analysis

Putamen seed centers of mass were chosen based on a prior report (50). Six seeds of radius 4mm were created in MNI152 space at 1×1×1mm resolution: Dorsal Rostral Putamen (DRP; ±25, 8, 6), Dorsal Caudal Putamen (DCP; ±28, 1, 3), and Ventral Rostral Putamen (VRP; ±20, 12, -3). Seeds were subsequently transformed to 3×3×3mm using nearest-neighbor resampling. The Pearson’s correlation of the average seed time-series and cleaned whole-brain EPI time-series was computed and subjected to Fisher’s r-to-z transform.

### Functional MRI analysis

To test the hypothesis that the RSFC pattern of the putamen differs between depressed adolescents with a history of suicide attempt and those with no such history, we performed two levels of analysis as described below.

### First-level analysis

Putamen seed centers of mass were chosen based on a prior report (50). Six seeds of radius 4mm were created in MNI152 space at 1×1×1mm resolution: dorsal rostral putamen (DRP; ±25, 8, 6), dorsal caudal putamen (DCP; ±28, 1, 3), and ventral rostral putamen (VRP; ±20, 12, -3). Seeds were subsequently transformed to 3×3×3mm using nearest-neighbor resampling. The Pearson correlation of the average seed time-series and cleaned whole-brain EPI time-series was computed and subjected to Fisher’s r-to-z transform.

### Group-level analysis

Whole-brain voxel-wise *t*-tests (one-sample) were conducted to identify regions where RSFC was significantly different from 0. These were thresholded at voxel-wise *p*=.001.

Minimum cluster sizes were determined by a Monte-Carlo method that accounts for the estimated smoothing by using permutation testing. This method has been shown to accurately control the false positive rate (51). The cluster-wise *p* value was set at.01 and the associated minimum cluster size was 2,754 μL (102 voxels).

Between-group (AT versus NAT) differences in each seed’s RSFC were assessed using whole-brain *t*-tests. These differences were constrained to lie within the mask showing significantly non-zero RSFC identified using the one-sample *t*-tests described above (voxel-wise threshold: *p* = 0.01). Correction for multiple comparisons was accomplished identically to the one-sample tests. The cluster-wise *p*-value was set at 0.05 yielding a minimum cluster size of 4,590 μL.

### Demographic and clinical measures analysis

Analyses were conducted using R (52) to assess between group differences and to determine how well matched the groups were. Welch *t*-tests or Wilcoxon tests were used to assess group differences in continuous variables and χ^2^ tests were used to assess group differences in categorical variables.

The Spearman’s correlation analysis was performed to assess brain-behavior correlations. Specifically, we analyzed associations between any putamen RSFC patterns and suicidal ideation severity as well as lethality of AT as measured by the C-SSRS within the AT group.

## Results

### Demographic and clinical measures

All participants were right-handed, and the groups were well-matched for IQ, socioeconomic status, age, gender, ethnicity, and pubertal stage, as well as clinical measurements, with significant differences reported only for Weschler Abbreviated Scale of Intelligence (Verbal and Full scales) (**Table 1**).

### Group differences in putamen resting-state functional connectivity

**Figure 1:**
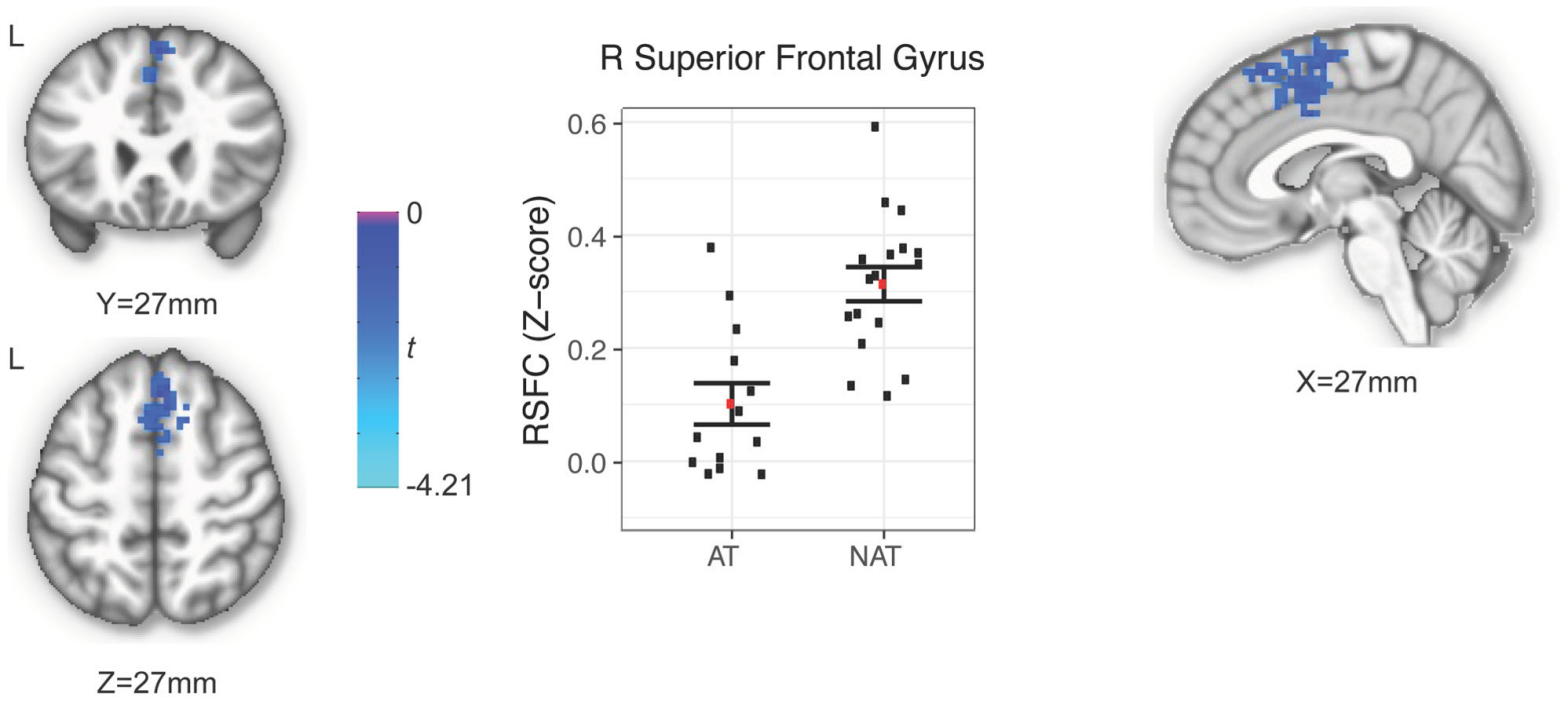
Region showing between-group difference of RSFC of the right dorsal rostral putamen.

Of the six putamen seeds, only the dorsal rostral putamen showed a statistically significant between-group difference in RSFC, with higher RSFC in the NAT (z=0.31) compared to the AT (z=0.1) group. The region identified occupied 9,477 μL with a peak voxel at (x=-2, y=-24, z=54) and extended bilaterally into the superior frontal gyrus and supplementary motor area (z=-2.94, *p* < 0.05; **Figure 1**).

### Brain-behavior correlations

The relationship between brain connectivity and features of suicidality was assessed by using Spearman’s correlations. No significant correlations were identified between any putamen RSFC patterns and suicidal ideation severity or lethality of AT as measured by the C-SSRS within the AT group (all *ps* > 0.1).

## Discussion

To our knowledge, the current study is the first to examine potential group differences of putamen RSFC in depressed adolescents with a history of suicide attempt relative to those with no such history. Our results suggest that putamen circuitry may play an important role in adolescent suicidality. Specifically, we found that the dorsal rostral region of the putamen had a statistically significant between-group difference in RSFC, with higher RSFC in the NAT compared to the AT group. The region extended bilaterally into the superior frontal gyrus and supplementary motor area. These findings are important to help advance the field of adolescent suicidality research as they improve our understanding of the brain circuitry associated with history of suicide attempt and may help guide therapies as discussed in more detail below.

The current study findings have a striking similarity with those from a recent study conducted by Wagner and colleagues (53) which suggests the existence of a heritable association with suicidal vulnerability. In that study, relatives of suicide victims exhibited two sub-networks of decreased RSFC compared with healthy subjects, one of which (*p* = .02) was composed of 21 nodes connected by 26 edges mainly located in the fronto-cingulo-striatal network, i.e., the bilateral putamen, bilateral anterior cingulate cortex, dorsomedial prefrontal cortex, bilateral supplementary motor area, right premotor cortex, bilateral thalamus, right superior temporal gyrus, and right hippocampus.

**Figure 2:**
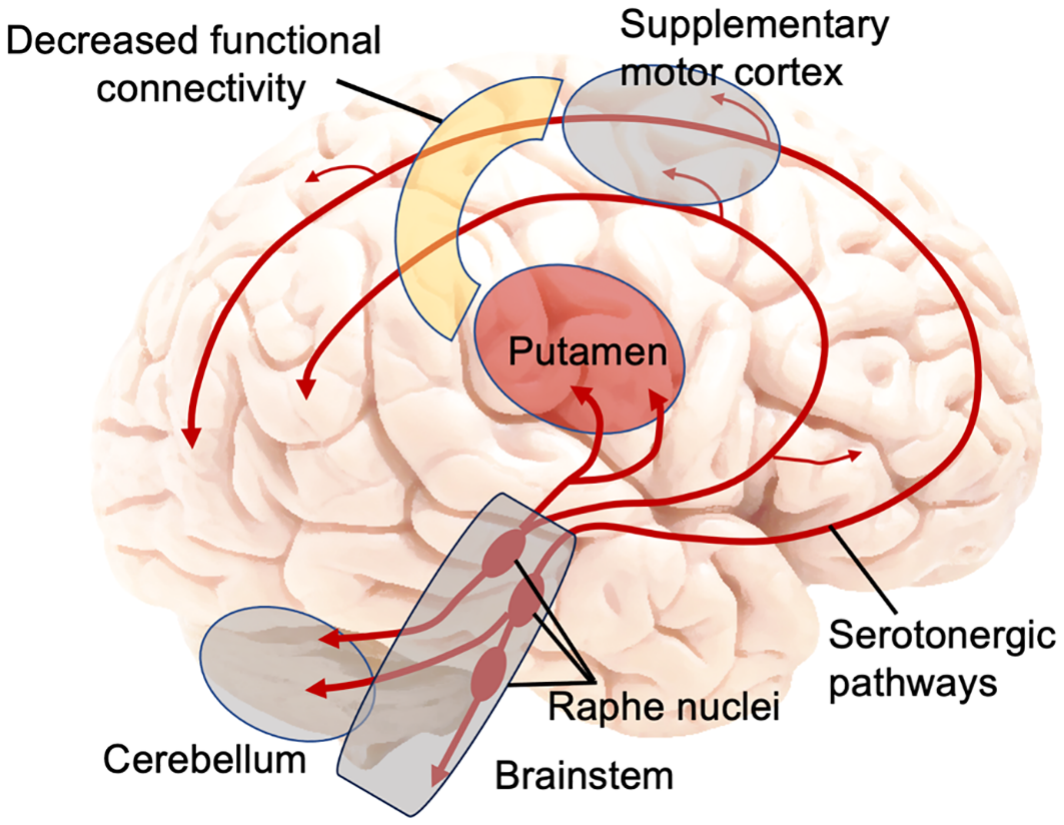
Main serotonin pathways in the brain and decreased functional connectivity between putamen and supplementary motor cortex in adolescent suicide attempters. Figure was constructed by the authors, adapted from (54).

Notably, the regions obtained in our study – largely located in the supplementary motor area – as well as the seeded putamen, are all located within the central serotonergic pathway in the brain (54) (**Figure 2**). Serotonin is produced in the raphe nuclei in the brainstem, from which serotonergic projections project to the striatum [including the putamen, to a larger degree than the caudate (15)] and neocortex (including the supplementary motor area) (54) (**Figure 2**). Collectively, these serotonergic systems play a critical role in mood, avoidance behavior, fear, and anxiety (54). The results of our study suggest that there may be a mismatch of the serotonin transport to putamen and to the supplementary motor area and the resulting reduced functional connectivity between the two areas in adolescents with a history of a suicide attempt.

There are several psychological constructs that are potentially linked to the aberrations found in this study, such as impulsivity and delay discounting. Whereas the sensory motor area (as an inhibitory structure) plays a critical role in exerting control over voluntary actions in situations of response conflict (55), impulsivity may relate to striatal gray matter volumes (56). In regard to adolescent suicide, Spirito and colleagues (57) concluded that impulsive aggression may be the mechanism through which decreased serotonergic activity is related to suicidal behavior (57). However, the link between impulsivity and suicidality has been questioned (58, 59). Unfortunately, we did not assess aggression or impulsivity in the current study and, thus, cannot contribute to this issue experimentally. It is also possible that a more sophisticated mechanism involving a social component is at play. For example, one recent study demonstrated that during peer exclusion and inclusion, youth with high suicidal ideation, including AT, showed significantly lower activity in precentral and postcentral gyrus, superior temporal gyrus, medial frontal gyrus, insula, and putamen compared to youth with lower suicidality (17). Finally, the well-documented fundamental involvement of the putamen in psychological pain and sadness (60) may represent the brain – behavior link of this dysregulation observed in those with AT.

### Theoretical and clinical implications

We would like to describe two examples of theoretical implications of our results. The obtained results suggest that the diathesis-stress model recently proposed by Mann and Rizk (7) (7) may be enhanced by adding putamen explicitly to their model, where serotonin dysfunction is implicated. The aforementioned study by Wagner and colleagues (53) further suggests that it is the diathesis—rather than the stress—part of the model that is linked to the fronto-cingulo-striatal network, given that relatives without mental illness showed this aberration. At the same time, reductions—and not increases of putamen volume—were observed in subjects with a family history of suicide compared with subjects who used a violent suicidal means, which suggests that some morphological variations in this structure may represent endophenotypes of suicidal vulnerability, while others may modulate action selection (61).

Our results can also help refine another model, specifically the Emotional paiN and social Disconnect (END) model of adolescent suicidality recently proposed by our group (62). This model is centered on two key neural circuits: (1) the emotional/mental pain circuit, and (2) the social disconnect/distortion circuit. In the original END model, the emotional pain circuit, consisting of the cerebellum, amygdala, and hippocampus, shows similar aberrations in adolescents with suicidal ideation as in AT (but to a smaller degree). The social disconnect circuit is unique to adolescent AT and includes the lateral orbitofrontal cortex, the temporal gyri, and the connections between them. As mentioned above, involvement of putamen in psychological pain and sadness has been well-documented (60). The current study results, in conjunction with prior literature, may warrant adding the putamen to the psychological pain circuit of the conceptual END model, along with the cerebellum – another important structure on the serotonergic pathway robustly linked to psychological pain (54, 60, 62) (**Figure 2**).

This study may have potential wider future clinical implications, provided routine application of MRI becomes widespread in adolescent psychiatric practice (63). For example, based on our results and the discussed models, it is possible that connectivity between putamen and the supplementary motor area will increase with treatment, and we suggest that, if confirmed, this connectivity may be used as a valuable biomarker of treatment efficacy and possibly prediction of treatment outcome. We note the lack of experimental evidence to date regarding the impact of treatment on increasing connectivity between these regions, and further research is needed to validate this possibility.

Results of this study should be interpreted in the light of limitations, including its retrospective nature and the modest sample size secondary to the difficulty of recruiting this particular population. Although the homogeneity of our sample is a study strength and depression is one of the strongest psychiatric risk factors for suicide, suicidal behaviors are nonetheless a transdiagnostic phenomenon; focusing solely on depressed adolescents precludes us from examining whether or not putamen-based RSFC patterns are present in adolescents with other psychiatric disorders who have a history of suicide attempt. Another important limitation is that we are not measuring serotonin directly. Positron emission tomography that is commonly used for such measurements is not typically performed in adolescents due to concerns of using radioisotopes in teens. Neurotransmitters other than serotonin (in particular, dopamine) as well as hormones may play a role in putamen activity and structure. Given evidence of serotonergic regulation of dopamine transmission, and the complex interactions that occur between these two neurotransmitter systems (64, 65), it may be the case that dopamine and/or serotonin dysregulation will prove to mechanistically explain potential links between striatal volume – specifically putamen volume - and suicidal vulnerability (66). For instance, work in an independent sample of adolescents has demonstrated that behavior on an implicit suicidal cognition task is related to volumes of the putamen and caudate in depressed adolescents (66) and typically developing youth (67). Both the putamen and caudate are rich in dopaminergic receptors and are involved in processes related to stimulus–action mappings (e.g., prepotent inhibition, motivational behaviors, etc.). The putamen and sensory motor area play a crucial role in the dopamine pathway in other diseases where behavior and movement execution are dysregulated (e.g., Parkinson’s disease, 68), suggesting that future study of suicidal vulnerability among adolescents should consider the role of both serotonin and dopamine. As mentioned earlier, in our study we did not assess impulsivity, psychological pain, or some other constructs that could have improved our understanding of suicidal behaviors in youth. Future directions would include measuring such constructs and testing refined theoretical models of suicide risk (such as our END conceptual model of adolescent suicide or those developed by Mann and Rizk).

In summary, the current study found compelling evidence of a relation between dorsal rostral striatum and bilateral between-group differences in connectivity with the superior frontal gyrus and supplementary motor area. Our findings of increased connectivity in the group without a suicide attempt compared to those with attempt history suggest that putamen circuitry may serve as an important biomarker associated with adolescent suicidality. Findings also suggest pathways of further inquiry related to testing conceptual models that incorporate dopamine and serotonin transmission.

## Data availability statement

The raw data supporting the conclusions of this article will be made available by the authors, without undue reservation.

## Ethics statement

The studies involving humans were approved by the institutional review boards of University of California San Diego, University of California San Francisco, Rady Children’s Hospital, and the county of San Diego. The studies were conducted in accordance with the local legislation and institutional requirements. Written informed consent for participation in this study was provided by the participants’ legal guardians/next of kin.

## Author contributions

OT: Writing – original draft, Writing – review & editing, Conceptualization, Methodology. TH: Conceptualization, Data curation, Formal analysis, Methodology, Writing – original draft, Writing – review & editing, Funding acquisition. CC: Conceptualization, Data curation, Formal analysis, Methodology, Software, Visualization, Writing – original draft, Writing – review & editing. SG: Methodology, Writing – original draft, Writing – review & editing, Conceptualization. RR: Writing – original draft, Writing – review & editing. SD: Writing – original draft, Writing – review & editing. JM: Writing – original draft, Writing – review & editing. TTY: Conceptualization, Funding acquisition, Investigation, Methodology, Project administration, Resources, Supervision, Writing – original draft, Writing – review & editing.
